# Pulmonary Neoplasms with Pleuropulmonary Blastoma-like Features in Two Young Labrador Retrievers: Clinicopathological, Molecular and Comparative Characterisation

**DOI:** 10.3390/vetsci13101111

**Published:** 2026-10-17

**Authors:** Louise van der Weyden, Bailey A. Francis, Damon R. Olson, Kris Ann P. Schultz, Stefano Di Palma, Tony Ryan, Kasia Kamieniarz, Maria Miguel-Garces, Layla Thompson, Rebecca Jones, Charlotte Nix, Sean Haugland, Ana L. Ortiz

**Affiliations:** 1 Wellcome Sanger Institute, Wellcome Genome Campus, Hinxton, Cambridge CB10 1SA, UK; lvdw@sanger.ac.uk (L.v.d.W.); bf14@sanger.ac.uk (B.A.F.); 2 Children’s Minnesota, Department of Pathology and Laboratory Medicine, 2525 Chicago Avenue, Suite B600, Minneapolis, MN 55404, USA; damon.olson@childrensmn.org; 3 Children’s Minnesota, Cancer and Blood Disorders, Children’s Minnesota Research Institute, 2530 Chicago Avenue South, CSC-175, Minneapolis, MN 55404, USA; krisann.schultz@childrensmn.org; 4 International PPB/*DICER1* Registry, Children’s Minnesota, Minneapolis, MN 55404, USA; 5 IDEXX Laboratories, Grange House, Sandbeck Way, Wetherby LS22 7DN, UK; stephano-dipalma@idexx.com; 6 Southern Counties Veterinary Specialists, Forest Corner Farm, Hangersley, Ringwood, Hampshire BN24 3JW, UK; tony.ryan@scvetspecialists.co.uk (T.R.); kasiaatmaca@gmail.com (K.K.); maria.garces@scvetspecialists.co.uk (M.M.-G.); 7 The Queen’s Veterinary School Hospital, University of Cambridge, Cambridge CB3 0ES, UK; ljt59@cam.ac.uk (L.T.); cd471@cam.ac.uk (C.N.); 8 North West Veterinary Specialists, Runcorn WA7 3FW, UK; becky.jones@nwspecialists.com; 9 Veterinary Pathology Group, 540 The Crescent, Stoke Gifford, Bristol BS16 1EJ, UK

**Keywords:** lung, pneumothorax, tumour, cystic, immunohistochemistry, vimentin, molecular, genome, *DICER1*, One Health

## Abstract

Pleuropulmonary blastoma (PPB) is a rare, paediatric, mesenchymal, pulmonary neoplasm that can be highly aggressive. To date, there are only two reports of PPB in animals. We describe the finding of pulmonary neoplasms with PPB-like features in two unrelated Labrador Retrievers. Both dogs presented with acute dyspnoea, pneumothorax and pulmonary lesions. Treatment consisted of emergency thoracostomy tube placement followed by lung lobectomy. At follow-up, one dog experienced no recurrence 20 months post-surgery, whereas recurrence developed in the second dog 2 years postoperatively. Histopathological examination revealed spindle-shaped neoplastic cells with blastematous-to-sarcomatous appearance, associated with cystic spaces lined by non-neoplastic respiratory epithelium. Immunohistochemistry showed that the neoplastic cells were positive for mesenchymal markers but negative for epithelial and neuroendocrine markers, with some tumour cells showing desmin positivity, supportive of myogenic differentiation, possibly compatible with rhabdomyoblastic differentiation. Genome profiling revealed a heterogeneous somatic landscape. No pathogenic somatic or germline *DICER1* variants were found. We compared these findings with those reported in human PPB. This is the first comprehensive analysis of pulmonary neoplasms with PPB-like features in animals, and reveals parallels with PPB in humans, further supporting the ‘One Medicine’ approach.

## Simple Summary

Pleuropulmonary blastoma (PPB) is a type of lung cancer that can be very aggressive and arises in the lungs, pleura or mediastinum of children. It is rare in humans, and there are only two existing reports of PPB in animals. Here, we describe the finding of pulmonary neoplasms with PPB-like features in two young unrelated Labrador Retrievers. Both dogs presented with sudden breathing difficulty and leakage of air from the lungs. Surgery was performed to evacuate the air and remove the mass. At 20 months post-surgery, one dog shows no symptoms of recurrence, whereas recurrence developed in the second dog 2 years postoperatively. Histopathology revealed spindle-shaped cancerous cells, some compatible with immature, primitive mesenchyme, associated with cystic spaces. On immunohistochemistry, cancerous cells were positive for mesenchymal markers but negative for epithelial and neuroendocrine markers, with some tumour cells showing desmin positivity. DNA sequencing revealed a heterogeneous somatic landscape, with no pathogenic *DICER1* variants identified. We compared these findings with those seen in human PPB. This is the first comprehensive analysis of lung cancer with PPB-like features in animals and reveals similarities with PPB in humans.

## 1. Introduction

Pleuropulmonary blastoma (PPB) is a rare and aggressive paediatric, pulmonary neoplasm. It is an embryonal (primitive mesenchymal) tumour of the lung and differs from pulmonary blastoma (PB) which is a sarcomatoid carcinoma (biphasic epithelial/mesenchymal) almost exclusively seen in adults [1,2]. PPB represents 0.2–0.3% of all human paediatric cancers; however, due to its rarity, it sometimes goes unrecognized and it has been suggested that the incidence rate of PPB may be underestimated [3]. It occurs mainly in children up to 7 years of age and is the most common primary lung tumour of infancy and early childhood [4]. In humans, PPB is classified into four histological subtypes, which represent a developmental continuum: type I (cystic), type II (cystic and solid) and type III (solid), with type Ir (regressed) PPB being a cystic lesion lacking a primitive malignant population [5]. Type I PPB typically presents in younger children, with a median age of 7 months, whereas types II and III are typically diagnosed in older children, with a median ages of 35 and 39 months, respectively [6]. Type Ir (regressed) PPB is seen in individuals from infancy through adulthood [6]. Accurate histological diagnosis is important as there can be progression from type I to types II/III, the latter of which are more aggressive, may recur or metastasize and are associated with a significantly poorer prognosis [5,7,8]. PPB patients typically have a germline and/or somatic pathogenic/likely pathogenic variant in the *DICER1* gene [9].

Primary pulmonary neoplasms are relatively uncommon in domestic animals. In dogs, they account for ~1% of all malignancies [10]. Canine pulmonary tumours are predominantly malignant, and ~85% are of epithelial origin, typically pulmonary papillary and bronchoalveolar carcinomas [11]. The current classification of pulmonary tumours in dogs [12] is much less extensive than the most recent list of lung tumours in the 2021 World Health Organisation (WHO) Classification of Thoracic Tumors in humans [13,14] and does not include a recognised veterinary counterpart to pleuropulmonary blastoma. To date, there have only been two reports of PPB in domestic animals. The first was in an equine foetus [15]. The second was in a young dog, and the study reported the computed tomography (CT) appearance and surgical management of a pulmonary neoplasm with features of PPB in a one-and-a-half-year-old dog [16]. In this report, we further characterise the findings in this dog, as well as detail a second case with similar clinical and histological features in another young dog. This study provides a comprehensive report encompassing clinical presentation, radiological findings, histological appearance, immunohistochemical profile, whole-exome sequencing (WES) results, treatment and outcome/follow-up of two unusual canine pulmonary neoplasms showing PPB-like features, that allows a thorough comparison with the findings of PPB in humans, in line with ‘One Medicine’.

## 2. Clinical Presentation

Case 1: A 3-year-old female neutered Labrador Retriever presented at a primary care practice with acute-onset dyspnoea and tachypnoea, following a 2-day history of increased respiratory effort. Thoracic Focused Assessment with Sonography for Trauma/Triage (T-FAST) did not detect any fluid in the pleural/pericardial spaces, haematology results were unremarkable, and blood gas analysis revealed mild acidosis (pH 7.303, normal range 7.31–7.45). The dog was referred to a tertiary care practice, where she was assessed later the same day.

**Figure 1:**
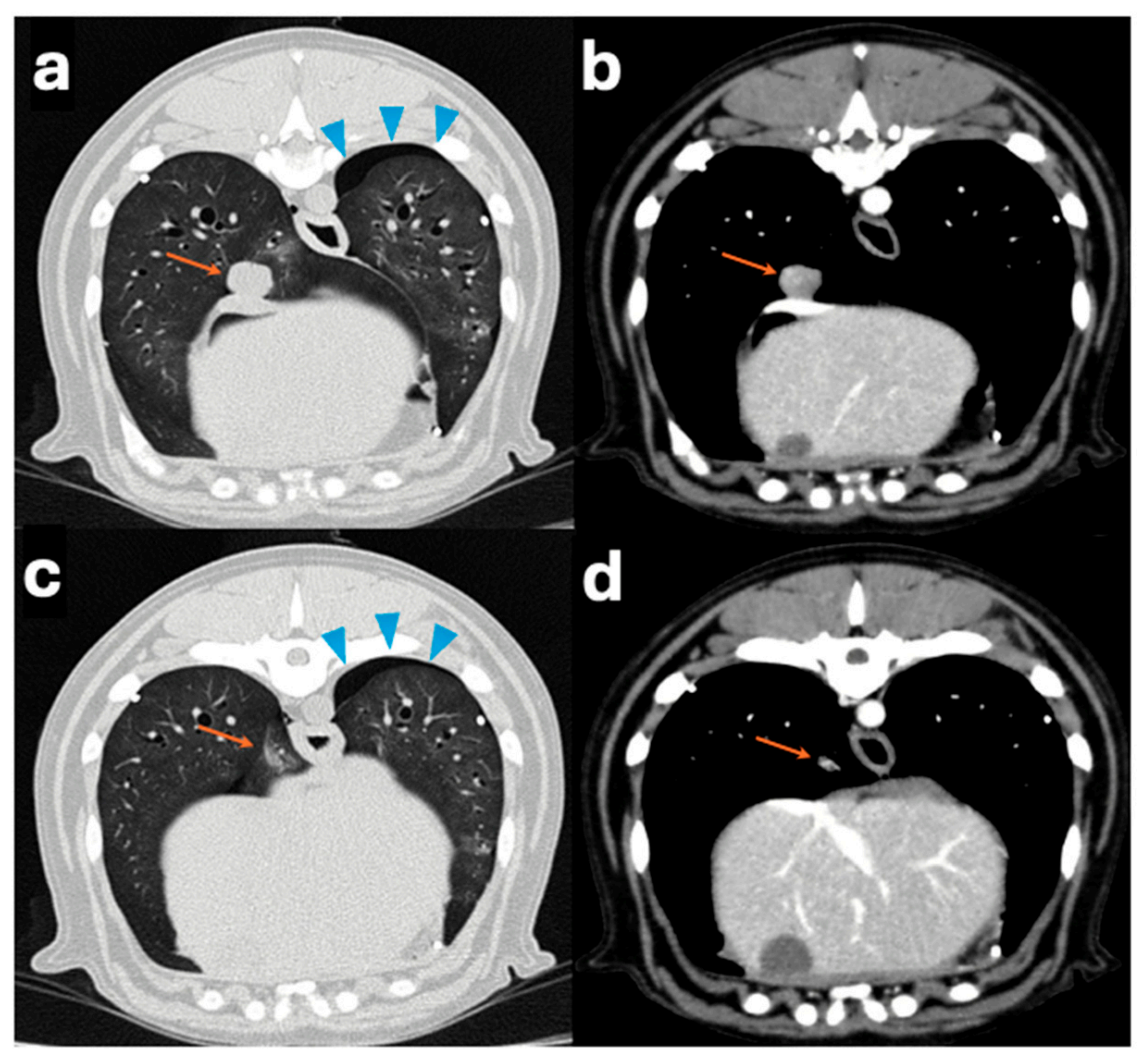
CT images of the caudal thorax of case 1. CT images are in the transverse orientation, including a lung window (**a**,**c**) and a soft tissue window post-contrast (**b**,**d**). (**a**,**b**) The CT images demonstrate a well-defined, heterogeneously contrast-enhancing soft tissue-attenuating nodule positioned within the accessory lung lobe (orange arrow), dorsal to the caudal vena cava. (**c**,**d**) The images demonstrate a second, smaller, heterogeneously contrast-enhancing soft tissue-attenuating nodule (orange arrow). (**a**,**c**) also illustrate free gas present dorsally within the pleural space of the thoracic cavity in the left hemithorax suggestive of a pneumothorax (blue arrowhead). There is a small, hyperattenuating chest drain present within both the right and left hemithoraces.

On presentation, the dog was bright, alert and responsive with a body condition score of 6/9. Auscultation revealed tachypnoea (respiratory rate of 60 breaths per minute) and bilaterally muffled lung sounds. A complete blood count was unremarkable, and serum biochemistry revealed elevated levels of serum amyloid A (SAA; 4.5 µg/mL; normal range 0–2.2 μg/mL) and phosphate (3.8 mmol/L, normal range 0.8 to 1.9 mmol/L). An *Angiostrongylus vasorum* antigen SNAP test was negative. Echocardiography showed sinus tachycardia (heart rate of 120 beats per minute) but no evidence of structural cardiac disease. Orthogonal thoracic radiographs revealed a bilateral tension pneumothorax. Bilateral thoracostomy tubes (MILA International, Inc., Hebron, KY, USA) were placed for emergency thoracocentesis, which resulted in immediate improvement in respiratory rate and effort. Following stabilisation, general anaesthesia and CT of the thorax were performed. This revealed a moderate residual bilateral pneumothorax and two soft tissue attenuating nodules within the right accessory lung lobe. The largest soft tissue nodule had very well-defined lobulated margins, measuring 1.7 cm in maximal diameter (Figure 1). The smaller of the two soft tissue attenuating nodules had more poorly defined wispy margins, measuring approximately 0.9 cm in diameter. Due to the poorly defined margins, the second nodule may have represented a region of rounded atelectasis. There was no evidence of any other thoracic or abdominal lesions.

The patient was monitored overnight in the intensive care unit, with thoracostomy tube drainage every 4 h. The following day, general anaesthesia and a median sternotomy were performed. Intraoperatively, a nodular lesion at the tip of the accessory lobe appeared ruptured, with subpleural gas bubbles, while the rest of the lungs appeared grossly normal. A right accessory lung lobectomy was performed using a 30 mm vascular linear stapler (PROXIMATE™ TX, Ethicon, Johnson & Johnson MedTech, New Brunswick, NJ, USA). The post-lobectomy air leak test was negative. The sternum was re-apposed using USP 0 polydioxanone cruciate mattress sutures encircling each sternebra. The deep pectoral musculature, subcutaneous tissue and skin were closed routinely. A 50 × 60 × 10 mm piece of lung containing one grey, moderately well-circumscribed nodule measuring 14 mm in diameter, with a cream, homogeneous cut surface, was placed into formalin and sent for routine processing for histopathological analysis.

The patient made a smooth recovery from surgery. The thoracostomy tubes were removed 2 days postoperatively, and the patient was discharged the following day. Medications at discharge were meloxicam (Metacam, Boehringer, 0.1 mg/kg, orally, q24 h for 14 days) and paracetamol/codeine (Pardale-V, Dechra, 400/9 mg, orally, 8 h for seven days). Two weeks later, the surgical incision was observed to be healing without complications, and the sutures were removed. Histopathology was consistent with a diagnosis of a neoplasm with pleuropulmonary blastoma-like features. Given the uncertain role of adjuvant therapy in veterinary patients due to limited published data, serial restaging was advised. The patient underwent three-view thoracic radiographs every three months for six months, followed by every 6 months thereafter. At 15 months postoperatively, there was no radiographic evidence of local recurrence or metastasis, and the patient remained free of clinical signs at 20 months post-surgery.

**Figure 2:**
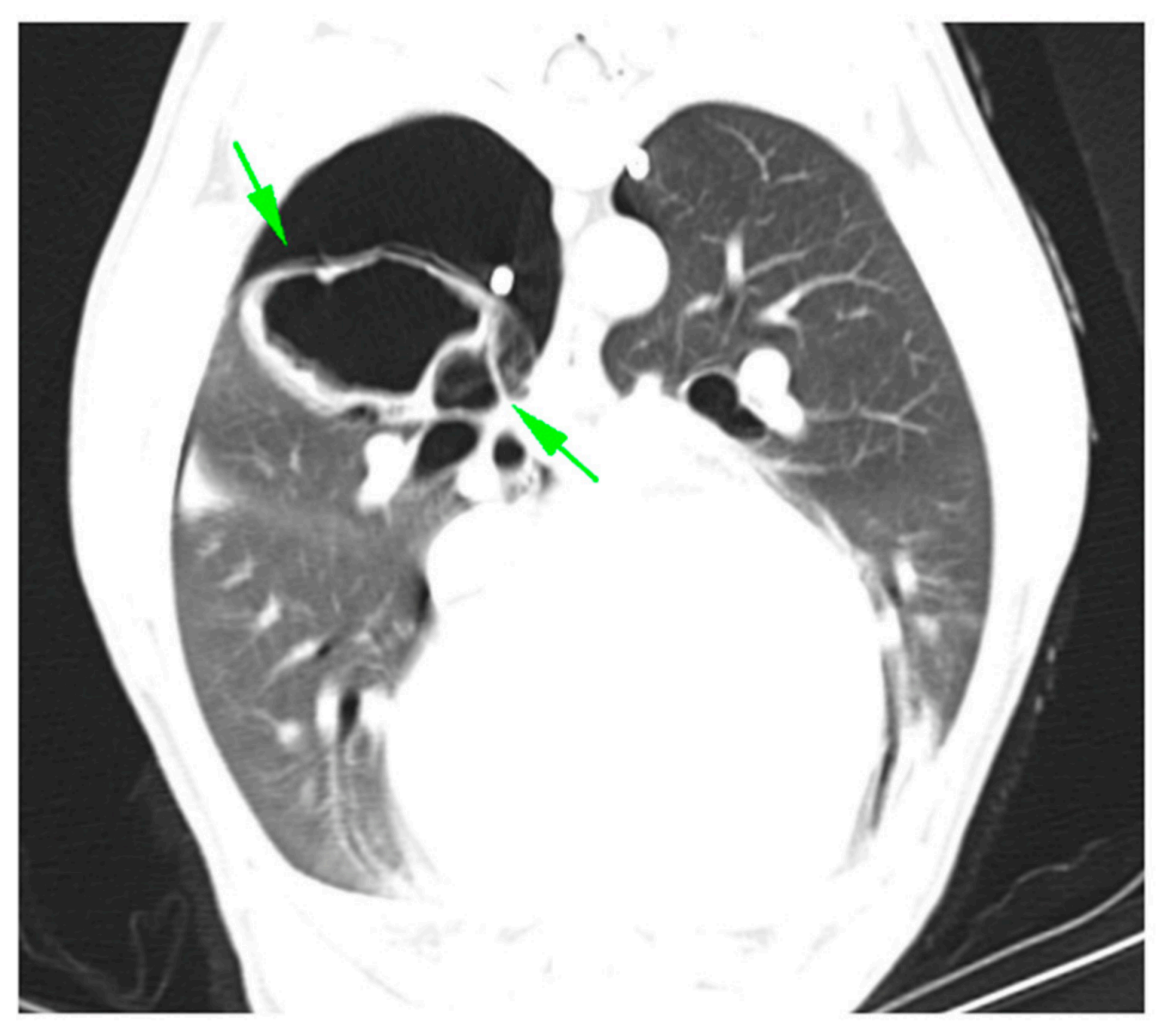
CT images of the caudal thorax of case 2. The CT image is in the transverse orientation and was taken post-draining. The arrows show the two cavitary lung lesions.

Case 2: As previously detailed [16], a one-and-a-half-year-old intact male black Labrador Retriever presented at a primary care practice following a 2-day history of a productive cough, dyspnoea and lethargy. Thoracic radiographs revealed a bilateral pneumothorax and a gas-filled cavitary lesion within the right caudal lung lobe. A thoracocentesis was performed, however, dyspnoea recurred a few hours later, and the dog was immediately referred to a tertiary care practice. At presentation, the dog was alert and responsive, with a body condition score of 4/9. The heart rate was 92 beats per minute, and auscultation revealed tachypnoea (a respiratory rate of 48 breaths per minute) with muffled heart and lung sounds. A complete blood count and blood serum biochemistry were performed, and the results were within normal limits. The diagnosis was recurrent pneumothorax, and an emergency thoracocentesis was performed. A CT scan of the chest revealed a large volume of gas in the right pleural space, consistent with right-sided tension pneumothorax, a small volume of gas in the left pleural space, and at least two cavitary lesions affecting the right cranial and right caudal pulmonary lobes. Bilateral thoracostomy tubes (MILA International, Inc., Hebron, KY, USA) were inserted, and air was evacuated from the pleural spaces (Figure 2). Considering the clinical history and CT findings, the diagnosis was spontaneous pneumothorax secondary to ruptured bullae or blebs. The patient was hospitalised, and intermittent evacuation of air was required overnight due to persistent pneumothorax.

The next day, an exploratory median sternotomy was performed and revealed a large necrotic mass and ruptured bulla affecting the right caudal lung lobe, and an intact bulla in the right cranial pulmonary lobe. The right middle, accessory and all the left lung lobes were grossly normal. A right cranial lung lobectomy was performed using a TA60 stapler (Coviden/Medtronic; Dublin, Ireland), and a right caudal lung lobectomy was performed using a TA90 stapler (Coviden/Medtronic). A beige, brown and grey piece of right caudal lung lobe, measuring 110 × 80 × 40 mm with a mass measuring 40 × 35 mm, and a piece of beige, brown and grey right cranial lung lobe measuring 120 × 80 × 25 mm, were placed into formalin and sent for routine processing for histopathological analysis. The air leak test post-lobectomy was negative, and new thoracic tubes were placed. The sternum, muscle, subcutaneous tissue and skin were subsequently serially reapposed with surgical sutures.

The recovery was without complications, and the post-operative analgesia was provided with bupivacaine (Bupivacaine Hydrochloride 0.5%, Mercury Pharma, 1 mg/kg every 8 h) administered via chest tubes in combination with oral meloxicam (Loxicom 1.5 mg/mL, Norbrook, 0.1 mg/kg once daily) and paracetamol with codeine (Pardale-V, Dechra, 15 mg/kg every 8 h). The thoracostomy tube was removed after three days and the patient discharged, while continuing analgesia with meloxicam. The patient was re-examined six weeks later; the surgical incision had fully healed, and all medications were discontinued. Chemotherapy regimens were discussed but not pursued. Restaging was performed four months postoperatively; haematological and biochemical profiles were within normal limits and CT of the neck, thorax and abdomen showed no evidence of recurrence. Repeat restaging was advised after 4–6 months; however, this was not pursued. At one year postoperatively, there had been no signs of respiratory compromise, suggesting no recurrence. However, almost 2 years after the initial surgery, CT identified a 1.5 cm pulmonary lesion within the accessory lung lobe with imaging characteristics similar to those of the original masses. Further surgery was planned; however, the dog was admitted a few days before the scheduled procedure because of a new pneumothorax. Repeat CT demonstrated changes interpreted as recurrent mass lesions involving the accessory and right middle lung lobes. At surgery, however, the right middle lung lobe appeared unaffected, and the pneumothorax was attributed to rupture of a recurrent mass arising from a remnant of the previously resected right cranial lung lobe. Grossly, the accessory lung lobe also appeared to contain a lesion similar in appearance to the original pulmonary masses, and this was also resected. The dog was doing well 4 months after the second surgery.

## 3. Histopathological Characterisation

Tissue samples taken from the lobectomies were fixed in formalin, and routinely processed, embedded and sectioned for histopathological evaluation. Sections (4 μm thick) were stained with haematoxylin and eosin (H&E) and examined by light microscopy by both veterinary and human pathologists. The cases were also discussed with a human paediatric oncologist.

**Figure 3:**
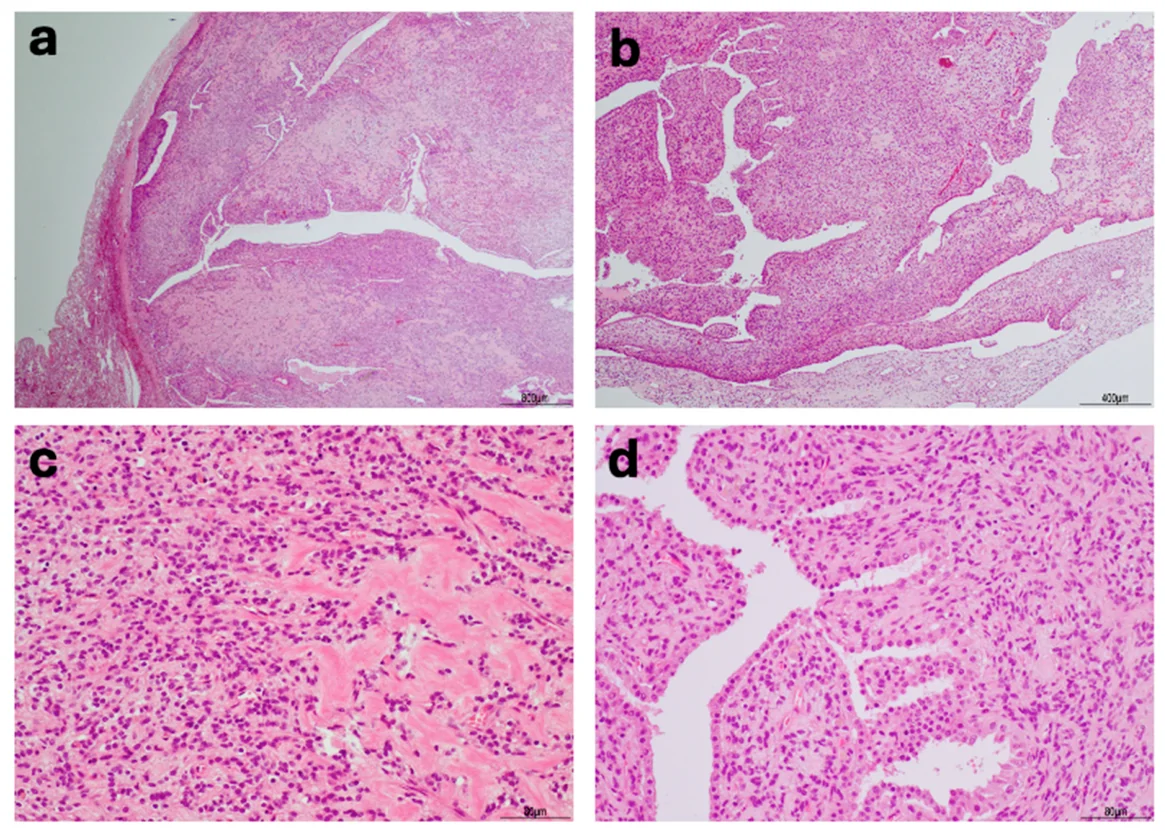
Histopathological features of the PPB-like lesion in case 1. (**a**) Low-power view showing a well-demarcated and densely cellular mass expanding and compressing the pulmonary parenchyma of the accessory lung lobe, ×20. (**b**) The neoplasm is composed of variably loosely arranged oval to spindle shaped mesenchymal cells with blastematous to sarcomatous appearance embedded in a fibrillar eosinophilic extracellular matrix and lined by a single layer of cuboidal to columnar epithelium, forming variably sized cystic structures, ×40. (**c**) Higher magnification of the mesenchymal component composed of oval to spindle-shaped cells with poorly distinct cell borders, scant eosinophilic cytoplasm, and ovoid to elongate nuclei, ×200. (**d**) Higher magnification of the non-neoplastic cuboidal to columnar epithelial cells lining cystic regions, ×200. Haematoxylin and eosin stain.

Case 1: Histopathological analysis of the accessory lung lobe sample revealed a well-demarcated, unencapsulated and densely cellular mass expanding the pulmonary parenchyma (Figure 3a). The mass was composed of loosely arranged sheets and streams of oval to spindle-shaped neoplastic cells with blastematous-to-sarcomatous appearance, in a moderately abundant, fibrillar and eosinophilic collagenous extracellular matrix, multifocally fenestrated by variably sized cystic structures lined by a single layer of ciliated to non-ciliated cuboidal to columnar or attenuated epithelium (Figure 3b). Similar epithelium was also observed lining large solid regions of oval to spindle-shaped cells multifocally forming discrete projections into optically clear spaces. The mesenchymal cells were oval to elongate to spindle, with poorly distinct cell borders, a scant eosinophilic cytoplasm and central ovoid to elongate nuclei with finely to coarsely stippled chromatin and often inconspicuous nucleoli (Figure 3c). The epithelial cells were cuboidal to columnar, with distinct cell borders, a moderate amount of eosinophilic cytoplasm and round nuclei with finely stippled chromatin and inconspicuous nucleoli (Figure 3d). Anisocytosis and anisokaryosis were mild in the epithelial cell population and moderate in the mesenchymal cell population, and the mitotic figure count was less than one per 2.37 mm^2^/10 high-power fields (×400). A few small lymphocytes and rare neutrophils and eosinophils were scattered throughout the neoplasm. At the surface of the mass, there were occasional regions of discontinuity of the epithelial lining associated with haemorrhage and a sparser collagenous matrix (consistent with oedema). Small numbers of lymphocytes and rare neutrophils were also present. The mass was compressing the adjacent pleura and pulmonary parenchyma, which exhibited mild to marked atelectasis, multifocal areas of mild to moderate haemorrhage and mild congestion of the vasculature. There was locally extensive to multifocal, moderate pleural and subpleural haemorrhage. In the planes of tissue examined, the mass appeared to have been fully excised, with tumour-free margins observed.

**Figure 4:**
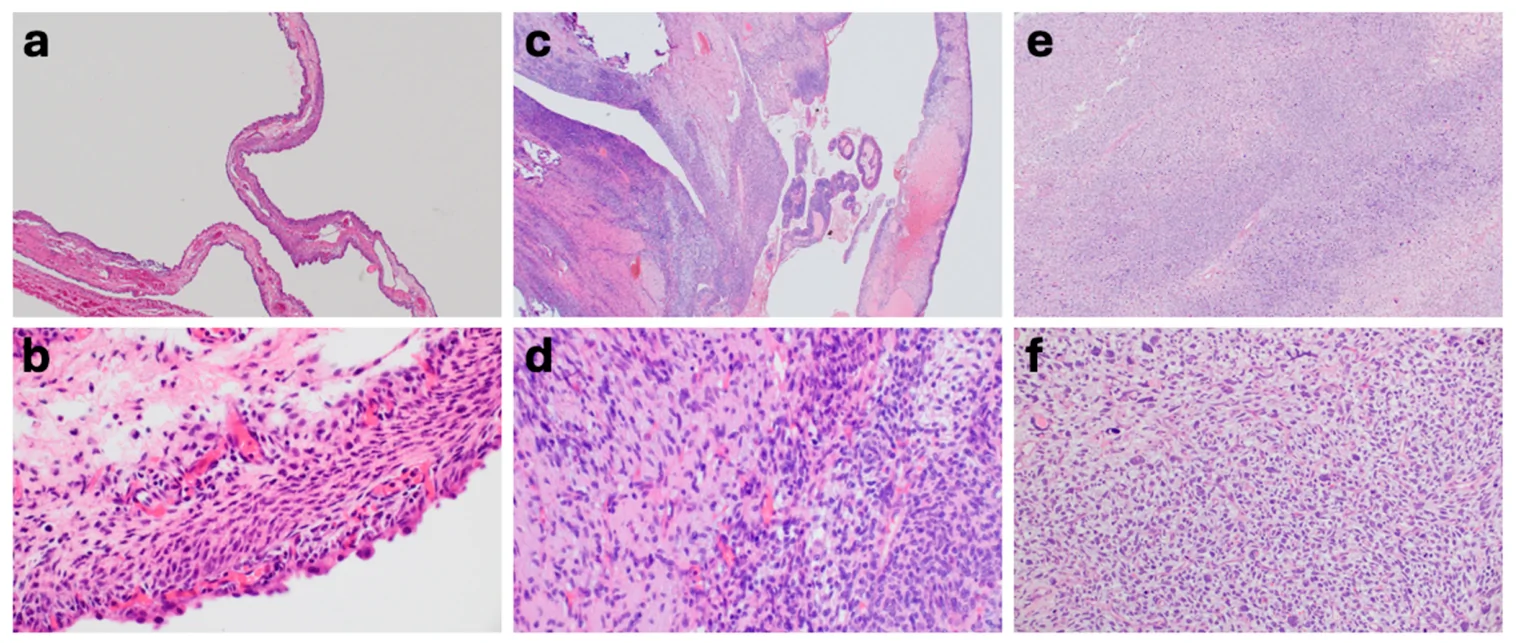
Histopathological features of the PPB in humans, types I-III. (**a**,**b**) Thin septa of a cystic type I pleuropulmonary blastoma with cambium layers of atypical spindle cells, ×40 (**a**) and ×400 (**b**). (**c**,**d**). Combined cystic and solid areas of a type II PPB with a primitive cambium-layer population of round and spindle cells expanding the septa into large solid areas, ×40 (**c**) and ×400 (**d**). (**e**,**f**) Solid type III PPB with markedly atypical primitive round and spindle cell populations embedded in a myxoid matrix with frequent scattered anaplastic cells and cytoplasmic hyaline droplets, ×40 (**e**) and ×200 (**f**). Haematoxylin and eosin stain. Image courtesy of Dr Damon R. Olson, Children’s Minnesota, Department of Pathology and Laboratory Medicine, reproduced with patients’ consent.

The diagnosis was consistent with an embryonal pulmonary neoplasm, with histopathology features resembling PPB in humans (Figure 4). Human PPBs are categorised based on gross and microscopic appearance, and although a similar classification is not available in veterinary medicine and the prognostic significance of these histological features in dogs is unknown, the mixed solid and, to a lesser degree, microcystic histological features of this mass appeared most similar to type II PPB. That said, it is acknowledged that this classification has not been validated in dogs and is being used solely as a comparative morphological reference in this instance. The human pathologist reviewing the case noted that the oval neoplastic cells considered to have a blastematous appearance were not as typically seen in paediatric cases, in which blastema cells are consistently smaller with a higher nuclear:cytoplasmic ratio and form a dense zone, or cambium layer, directly beneath the benign epithelial surface lining of the cystic regions [1]. Additionally, the collagenous stroma was considered more abundant when compared to paediatric PPB. Considering the presence of areas of discontinuity and haemorrhage superficially within the mass, rupture of the mass likely contributed to pneumothorax, and the atelectasis observed in the non-affected lung is consistent with the history of pneumothorax.

**Figure 5:**
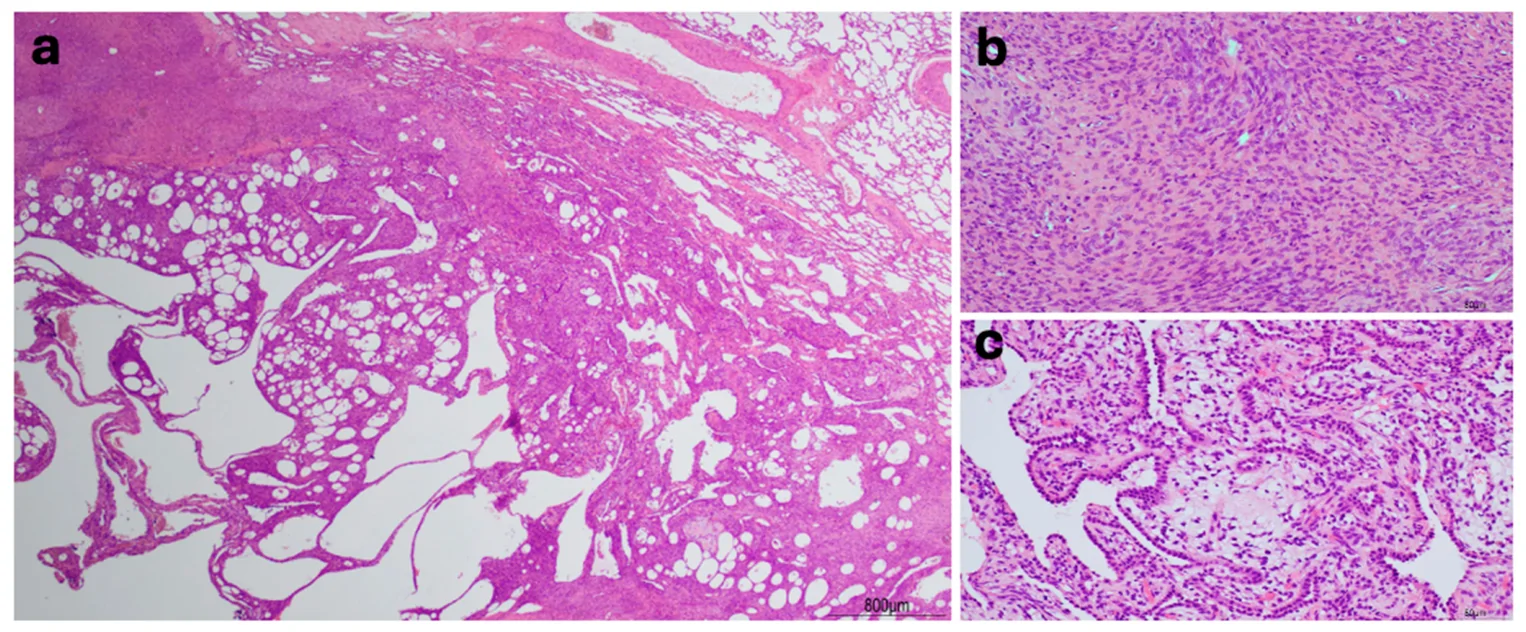
Histopathological features of the PPB-like lesion in case 2. (**a**) Lung tissue containing a lesion characterised by multiple cystic structures, with adjacent densely cellular regions, ×20. (**b**) Higher magnification of the mesenchymal component characterised by densely arranged elongate to spindle-shaped neoplastic cells with indistinct cell borders, eosinophilic cytoplasm and ovoid to elongated nuclei within a collagenous stroma, ×200. (**c**) Cystic structures lined by a single layer of cuboidal to attenuated non-neoplastic epithelial cells and underlying elongate to spindle cell proliferation on a looser eosinophilic to amphophilic collagenous stroma, ×200. Haematoxylin and eosin stain.

Case 2: Histopathological analysis of both the cranial and caudal lung lobe samples revealed multiple cystic areas, some discontinuous, in a multifocal to coalescing distribution expanding the lung parenchyma, with adjacent densely cellular, moderately to poorly demarcated areas of cellular proliferation (Figure 5a). Some of these areas comprised solid, densely arranged neoplastic oval to elongate to spindle-shaped cells within a dense collagenous stroma, admixed with empty lumina lined by a single layer of ciliated to non-ciliated cuboidal or attenuated cells. Other regions contained multiple variably sized cystic lumina, formed by trabeculae and papillary projections of loosely arranged spindle cells, also lined by cuboidal to attenuated cells. The oval to elongate to spindle cells had variably distinct cell borders, scant eosinophilic cytoplasm, and a central ovoid to elongated, frequently hyperchromatic nucleus (Figure 5b). These cells were multifocally embedded within a looser fibrillar eosinophilic to amphophilic stroma and multifocally admixed with small to moderate numbers of lymphocytes and plasma cells. The cuboidal epithelial cells lining lumina and cystic areas had distinct cell borders, a moderate amount of eosinophilic cytoplasm, and round nuclei (Figure 5c). Anisocytosis and anisokaryosis were mild in the epithelial cell population and moderate to marked in the mesenchymal cell population, and the mitotic figure count was one per 2.37 mm^2^/10 high-power fields (×400). Low to moderate numbers of lymphocytes and plasma cells were present throughout the lesion. Occasional moderately sized areas of necrosis and small accumulations of basophilic material consistent with mineral were present within the lesions. The adjacent parenchyma contained frequent large arteries with moderate to marked hypertrophy of the tunica media, frequently distended bronchi and bronchioles, areas of atelectasis, as well as mild to moderate multifocal infiltrates of neutrophils with fewer small lymphocytes, plasma cells, occasional macrophages and haemorrhage. Bronchioles appeared to show epithelial hyperplasia, and occasional adjacent alveoli exhibited pneumocyte type II hyperplasia. In the right cranial lung lobe, shaved tissue borders did not contain neoplastic proliferation in the planes of tissue examined. In the right caudal lung lobe, the neoplastic proliferation extended to shaved tissue borders, and complete excision was not confirmed.

The diagnosis was consistent with a pulmonary neoplasm with features of PPB in humans, along with bullae formation, necrosis, arterial wall hypertrophy, interstitial pneumonia and atelectasis. The neoplasm had both solid and cystic components, with extensive solid areas of spindle cell proliferation, and thus was considered to fall between types II/III using the human classification system as a comparative morphological reference (acknowledging that it has not been validated in dogs). Similarly to case 1, one of the human pathologists reviewing the case highlighted that the oval neoplastic cells were not as typical blastemal cells seen in paediatric cases in some areas and demonstrated an unusual infiltrative pattern into the alveolated lung interstitium rather than a more typical pushing border.

The recurrent pulmonary mass at the previous surgery site in the cranial lung lobe exhibited histomorphological features identical to those of the original tumour, supporting recurrence of the PPB-like neoplasm. The neoplasm extended to the shaved surgical borders, indicating incomplete excision. Despite the imaging appearance of changes affecting the accessory lung lobe, and the intraoperative appearance of a mass lesion affecting this lobe, neoplastic changes were not evident histopathologically.

## 4. Immunohistochemical Characterisation

To further characterise these lesions, immunohistochemistry (IHC) was performed to evaluate the expression of cytokeratin (1:100, pan-cytokeratin, clone MNF116; Agilent Dako, Stockport, UK), vimentin (1:500, clone V9, Agilent Dako), smooth muscle actin (SMA; 1:100, clone 1A4; Agilent Dako), desmin (1:100, clone D33; Agilent Dako), synaptophysin (1:50, clone DAK-SYNAP; Agilent Dako), and chromogranin A (1:500, clone DAK-A3; Agilent Dako). Following dewaxing, antigen retrieval was performed using a PT Link (Agilent Technologies, Manchester, UK) with EnVision™ FLEX Target Retrieval Solution, High pH (Agilent Technologies). Sections underwent heat-induced epitope retrieval according to the manufacturer’s protocol, including controlled pre-heating, high-temperature incubation, and gradual cooling within the PT Link system. Sections were then blocked with 2.5% normal horse serum (Vector Laboratories, Prestwich, UK) and incubated with the primary antibodies overnight at 4 °C. After washing, a secondary conjugated antibody (ImmPRESS™ Horse Anti-Mouse IgG; Vector Laboratories) was applied according to the manufacturer’s instructions. Immunoreactivity was visualised using the ImmPACT™ DAB Peroxidase Substrate Kit (Vector Laboratories). Sections were counterstained with Mayer’s haematoxylin. Positive control tissues were canine small intestine for vimentin, desmin and SMA; canine parathyroid gland for chromogranin; canine adrenal gland for synaptophysin; and canine stomach for cytokeratin. Negative controls consisted of omission of the primary antibody. Immunohistochemical staining was assessed by semiquantitative visual estimation.

**Figure 6:**
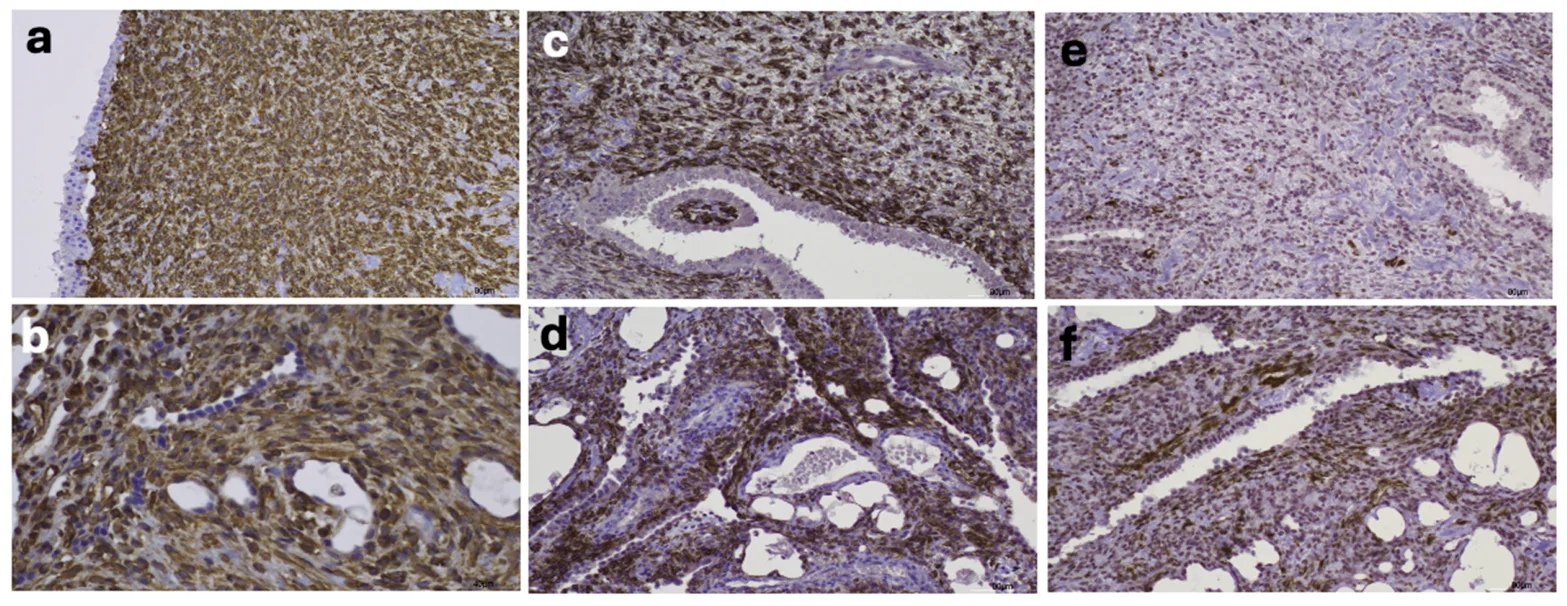
Immunohistochemical features of PPB-like lesions in cases 1 and 2. (**a**,**b**) Strong cytoplasmic immunolabelling for vimentin in most neoplastic oval to elongate to spindle shaped cells, confirming mesenchymal differentiation, ×200 (**a**) and ×400 (**b**). (**c**,**d**) Frequent strong cytoplasmic immunolabelling for desmin in neoplastic spindle cells, supporting myogenic differentiation of the mesenchymal component, ×200. (**e**,**f**) Less frequent smooth muscle actin (SMA) cytoplasmic immunolabelling in the neoplastic cells (estimated to be present in 5–10% of the neoplastic spindle cells in case 1 and 20–25% of these cells in case 2), ×200. Immunohistochemistry with haematoxylin counterstain.

As detailed in Table 1, positive immunolabelling for vimentin was seen in most of the neoplastic oval to elongate to spindle cells for both cases, supporting a mesenchymal phenotype (Figure 6a,b).

The same cell population did not show any immunolabelling with the epithelial cell marker cytokeratin. The preferential immunolabelling of the tumour cells for desmin (Figure 6c,d), rather than SMA (Figure 6e,f), supports myogenic differentiation of some of the tumour cells down the skeletal muscle lineage and is compatible with possible rhabdomyoblastic differentiation, although myogenin or MyoD1 IHC would be needed to confirm this.

The neoplastic cells in both cases were immunonegative for the neuroendocrine cell/tumour markers synaptophysin and chromogranin.

Additional immunohistochemistry was not performed on the recurrent neoplasm.

## 5. Molecular Characterisation

To characterise the molecular landscape of both canine pulmonary neoplasms, we performed WES on the tumour and adjacent normal formalin-fixed, paraffin-embedded (FFPE) tissue samples from both dogs. Genomic DNA was extracted using the QIAamp DNA FFPE Tissue Kit (Qiagen; Manchester, UK) according to the manufacturer’s instructions. Sequencing libraries were prepared from the FFPE-extracted DNA as previously described [17], pooled (8-plex) in an equimolar fashion and then hybridized overnight with SureSelect CD Canine Exome bait sets (Agilent Technologies) [18]. The multiplexed samples were paired-end sequenced using the NovaSeq platform (Illumina, Great Abington, Cambridge, UK) to generate 101-bp reads. The sequencing reads were aligned to the CanFam3.1 reference genome [19] using BWA-MEM, and PCR duplicates were marked using Biobambam2 bammarkduplicates, as previously detailed [17]. All samples (tumour and normal) had ≥20× coverage of at least 80% of the bait regions. Somatic variants, somatic copy number alterations and germline variants were identified using the algorithms previously detailed [17]. In brief, somatic variants were identified using cgpCaVEMan (v1.15.2) [20] for single-nucleotide variants (SNVs) and cgpPindel (v.3.10.0) [21] for insertions/deletions (indels). Variant calls from cgpPindel were filtered so that only variants with a variant allele fraction (VAF) ≥0.1 were retained. Any indel in which both the reference and alternate alleles were >25 bp was only retained if it had a coverage depth >20× in both the tumour and the matched normal samples and a VAF of > 0.25. Finally, only variants overlapping the WES bait target regions that passed both the cgpCaVEMan and cgpPindel filter flags were retained for downstream analysis. The set of candidate somatic variants was then compared to two public datasets of whole-genome-sequenced dogs: Dog10K (1920 dogs, including 6 Labrador Retrievers) [22] and the Dog Genome Project (DGP) (668 dogs, including 24 Labrador Retrievers) [23]. The Dog10K panel uses the newer CanFam4 reference genome assembly [24], so variant positions were converted to CanFam3.1 using BCFtools liftover (v1.22) [25]. Any variant that had a global population allele frequency of >1% in either panel was classified as a putative germline variant and was removed. After filtering, the Ensembl (v104) Variant Effect Predictor (VEP) [26] was used to predict the consequences of base changes and indels on proteins. Somatic protein-altering variants were found in four genes in case 1 (Table 2) and in five genes in case 2 (Table 3). The heterogeneous nature of the tumours is highlighted by an absence of overlap between the mutated genes in the two cases.

In humans, PPB somatic *DICER1* variants occur in almost all tumours (>90%) [27,28], predominantly within the RNase IIIb domain and involving one of five common “hotspot” locations [28]. Therefore, reads aligning to canine *DICER1* from all samples were manually inspected, and a single somatic variant was identified in the tumour sample of case 2, specifically c.3091C>T (p.Gln1031Ter; Chr8:63,990,129 G>A). This is a nonsense variant, predicted to truncate the protein, and the equivalent human residue (also Gln1031) is upstream of both RNase III domains. When considering non-duplicate reads, five out of 169 (3%) reads carried the alternate allele in the tumour sample of case 2 compared to 0 out of 174 reads in the matched normal. However, likely due to its low VAF, this variant was not called by cgpCaVEMan. Additionally, low-VAF C>T variants are a typical signature of FFPE deamination [29]. Therefore, we cannot definitively state that this is a subclonal *DICER1* variant, as it could equally just represent an FFPE artefact.

Somatic copy number alterations (CNAs) were identified using CNVkit (v0.9.10) [30]; however, there were no CNAs that exceeded the thresholds for amplification (log2(FC) > +0.32) or deletion (log2(FC) < −0.4) (including both focal and whole-chromosome deletions and/or amplifications).

Sequencing of matched normal tissue for both cases allowed the identification of putative pathogenic germline variants that may have predisposed these dogs to develop these pulmonary neoplasms with PPB-like features. Germline variant calls were generated with GATK HaplotypeCaller (v4.2.6.1) [31]. Raw variant calls were marked using GATK’s VariantFiltration and filtered using variant-type-specific criteria, following the GATK recommendations. SNVs were removed if QD < 2, QUAL < 30, SOR > 3, FS > 60, MQ < 40, MQRankSum < −12.5 or ReadPosRankSum < −8. Indels were removed if QD < 2, QUAL < 30, FS > 200 or ReadPosRankSum < −20. For both variant types, a minimum depth of 20 and a minimum genotype quality of 20 were also required. Only variants passing all filters that overlapped the WES bait target regions were analysed further. Variants were annotated using the Ensembl Variant Effect Predictor (v104, CanFam3.1). To reduce false-positive germline calls caused by technical artefacts, a further set of filters was applied. Insertion variants whose sequence mirrored the exon sequences of the same gene were removed, as gene retrocopies are common and are highly polymorphic in dogs [32], and reads derived from retrogene copies of a gene can align to their parent gene and be misreported as germline insertion sequences. Indels in low-complexity or highly repetitive sequences were also removed. The set of candidate germline variants was then compared to the Dog10K and DGP canine whole-genome sequencing panels (outlined above). After conversion of the Dog10K panel to CanFam3.1 coordinates, any CanFam3.1 variants whose alternate allele is the reference base in CanFam4, which typically represent errors or rare alleles in the CanFam3.1 reference, were removed. As PPB-like tumours are extremely rare in dogs, any candidate germline variants with >5% population allele frequency in either panel were classified as benign and were removed. Variants carried by any Labrador Retriever in either dataset were also excluded as breed-specific polymorphisms. Variants passing all criteria are reported as germline loss-of-function variants of unknown significance. There were seven such variants, all observed in case 2 (Supplementary Table S1).

In human patients, germline variants in *DICER1* are observed in the majority of PPB cases (ranging from 53% to >70%) [8,9,33,34,35]. No rare *DICER1* germline variants (<1% population allele frequency compared to Dog10K/DGP) were identified in either dog. The only protein-altering *DICER1* mutation was the missense variant p.Ala1395Thr, which was heterozygous in case 2. This variant is common in the dog population (Dog10K: 1099/3840 alleles (28.6%), DGP: 366/1334 alleles (27.4%)), as well as in Labrador Retrievers (Dog10K: 8/12 alleles (66.7%), DGP: 18/48 alleles (37.5%)) and is therefore too prevalent to be a likely cause of the disease.

Additional molecular testing was not performed on the recurrent neoplasm. A summary of the clinical, follow-up, histopathological, immunohistochemical and molecular findings for both cases is provided in Table 4.

## 6. Discussion

In humans, the clinical presentation of PPB is usually one of non-specific respiratory symptoms such as difficulty breathing/respiratory distress (types I-III) and thus can mimic common paediatric illnesses such as pneumonia [34,36] which may potentially delay diagnosis. Additional symptoms such as fever, malaise and weight loss may be seen in type II/III PPB [36]. This mirrors the clinical presentation in these two dogs, with both showing acute-onset dyspnoea and tachypnoea, and case 2, which was diagnosed as type II/III, also displaying increased symptoms, including lethargy and coughing up phlegm. Additionally, the relatively early age at presentation, together with the distinctive histomorphological features, also invites comparison with human pleuropulmonary blastoma, which predominantly affects children but has rarely been reported in young adults [6]. PPB typically involves the lung and pleura, and a CT scan may show a pneumothorax together with a purely cystic, purely solid or mixed cystic and solid lesion, consistent with the observations in these two dogs. However, whilst radiologic features may increase the clinical suspicion for PPB, a diagnosis generally can only be made by histological analysis [5].

PPB is typically histologically heterogeneous, which can make the diagnosis challenging, depending on the experience of the pathologist and sampling of the lesion; the pleomorphic appearance of PPB can make the diagnosis from a small, inadequate biopsy very difficult [7]. For example, detailed histological re-examination of 49 type I cases found a wide variation of cellular components from one septal area to another, and whilst virtually all tumours had populations of small, primitive cells beneath the epithelial surface, these neoplastic cells ranged from being located in a small area of the cyst wall to being diffusely spread throughout well-defined cambium layers [7]. Similarly, the septal stroma was found to range from negligible to dense, acellular collagen, with some primitive neoplastic cells accompanied by differentiated rhabdomyoblasts or small nodules of immature cartilage, yet other cases showed no evidence of differentiation [7]. Type II and III PPB are typically characterised by blastematous islands associated with spindle-shaped cells that range from undifferentiated to various stages of malignant differentiation, including rhabdomyosarcoma, chondrosarcoma and liposarcoma [37]. The canine cases in this report showed cystic areas and a cellular mass expanding the pulmonary parenchyma that was composed of oval to elongate to spindle-shaped neoplastic cells of mesenchymal origin, as confirmed by their vimentin immunopositivity and epithelial marker immunonegativity, consistent with IHC results seen in human PPB [5,35,38,39]. Similarly, human PPB cases have shown rhabdomyoblasts being immunopositive for desmin [5,35,38], consistent with that seen in these two dogs. However, it is important to note that epithelial–mesenchymal transition is not necessarily disease-specific and can occur during epithelial remodelling in response to pathogens [40,41].

The European Cooperative Study Group for Pediatric Rare Tumours (EXPeRT) within the European Union-funded project PARTNER (Paediatric Rare Tumours Network—European Registry) highly recommends the diagnostic workup of a suspected type II or III PPB include a review of the histological slides by a pathologist with experience in paediatric tumours and especially in PPB and/or paediatric sarcoma [37]. It is notable that ~20% of suspected PPB cases referred to the International Pleuropulmonary Blastoma/*DICER1* Registry for review are incorrectly diagnosed [42]. Thus, in keeping with the recommendations for human PPB, but acknowledging possible species differences in the presentation and histopathology of similar disease conditions, the two cases in this report were reviewed by an experienced respiratory pathologist (case 2, DD) and by an experienced paediatric pathologist who serves as a central pathology reviewer for the International Pleuropulmonary Blastoma/*DICER1* Registry (D.O.). The cases were also discussed with a paediatric oncologist from the International Pleuropulmonary Blastoma/*DICER1* Registry (K.A.S.). It was noted that the oval neoplastic cells considered to have a blastematous appearance in these canine neoplasms were not as typically seen in paediatric cases, in which blastema cells are consistently smaller and more intensely basophilic and form a dense thick zone directly beneath the benign epithelial surface lining of the cystic regions [1]. The collagenous stroma was also considered more abundant when compared to paediatric PPB. It was also highlighted and agreed upon by both the veterinary pathologist and the paediatric pathologist that the mesenchymal cell population in case 2 exhibited increased pleomorphism when compared to case 1. Additional differential diagnoses were considered, including inflammatory myofibroblastic tumour, myofibrosarcoma, leiomyosarcoma, pulmonary synovial sarcoma (PSS) and congenital peribronchial myofibroblastic tumour. However, the morphology of the neoplasms in the present report does not conform well to these recognised entities. They lack the prominent inflammatory component characteristic of inflammatory myofibroblastic tumour (IMT). They do not exhibit the relatively uniform spindle and fascicular architecture expected in myofibrosarcoma or leiomyosarcoma, and prominent cystic regions lined by non-neoplastic epithelial cells would not be common in these neoplasms. While the mesenchymal stroma was multifocally dense, it frequently exhibited a blastematous-to-sarcomatous appearance on a variably loose matrix, rather than the uniform herringbone fascicles of PSS. The epithelial lining was well-differentiated and multifocally ciliated, suggesting entrapped respiratory epithelium rather than the malignant glandular differentiation of biphasic PSS. Furthermore, IHC results supported a mesenchymal phenotype of the spindle cell population (vimentin-positive, cytokeratin-negative) with heterologous myogenic differentiation (desmin-positive and SMA-positive), which is observed in PPB but typically absent in PSS. Frequent immature cartilage islands as expected in peribronchial myofibroblastic tumour are not a prominent feature in these canine cases, with the few cartilage islands present considered to represent entrapped pre-existent bronchial cartilage. While ALK testing, as performed in humans [43], and myogenin or MyoD1 IHC would have been helpful to exclude IMT and PSS, these ancillary diagnostic tests were not available through our laboratories.

Unlike human medicine, where the current WHO Classification of Thoracic Tumours recognises PPB as a distinct embryonal pulmonary neoplasm, there is currently no recognised canine pulmonary tumour that closely corresponds to this entity. Existing veterinary classifications describe primary pulmonary mesenchymal tumours, including osteosarcoma, chondrosarcoma and undifferentiated sarcomas, but none encompass the combination of clinicopathological, immunophenotypic and molecular features observed in the present cases. Although the tumours described here do not fulfil all diagnostic criteria for human PPB, their oval to spindle-shaped mesenchymal component with a non-neoplastic respiratory epithelial lining and cyst formation, occurrence in unusually young dogs and clinicopathological features more closely resemble those of human PPB. Recognition of similar cases may help determine whether these tumours represent a previously unrecognised entity within canine pulmonary neoplasia.

Nearly all cases of PPB in humans are associated with somatic and/or germline variants in *DICER1*, a gene encoding an RNase III-family endoribonuclease that is essential for the generation of small noncoding regulatory RNAs (which cleaves precursor microRNAs into active miRNA). PPB patients typically have biallelic pathogenic/likely pathogenic variants in *DICER1*. Germline heterozygous loss-of-function variants in *DICER1* have been described in familial PPB [44], also known as *DICER1*-related tumour predisposition, an autosomal-dominant condition which confers an increased risk of a range of benign and cancerous tumours, including PPB. Retrospective analyses of pathogenic germline *DICER1* variants in PPB patients have reported varying levels of prevalence, ranging from 53–66% in individual cohorts [8,33,34,35] to >70% in the PPB/*DICER1* Registry [9]. Analysis of the Registry samples identified three categories of PPB patients, based on their *DICER1* variant status: those with inherited or *de novo* germline loss-of-function *DICER1* variants (>70% patients), those with no germline variant but who are mosaic for predisposing *DICER1* variants (~10% of patients), and those with no predisposing germline or mosaic variants but who have somatic biallelic *DICER1* variants (typically affecting the RNase IIIb domain of the protein) [9]. Although exceedingly rare, there have been a few reports of PPB with an absence of either germline or somatic *DICER1* variants [27,45]. In these cases, it was suggested that low-level *DICER1* mosaicism may have been present but was not detected [9]. For the dogs in this case report, we did not identify a somatic *DICER1* variant using our standard somatic calling algorithm. Manual inspection of the sequencing reads did reveal a possible *DICER1* nonsense variant in case 2; however, the variant was not reported by the somatic variant calling pipeline, and because low-VAF C>T mutations are a typical signature of FFPE deamination, we cannot rule out the possibility of an FFPE-induced sequencing artefact. We also identified a germline *DICER1* variant in case 2; however, the high frequency of this variant both within the dog population as a whole and within the Labrador Retriever population (based on Dog10K and DGP databases), suggests it is unlikely to be causative. Thus, it may be that very low-level *DICER1* mosaicism was undetected, or that PPB-like neoplasms in dogs arise through different genetic processes from those in humans.

Whole-exome sequencing (WES) of 15 type II/II PPB tumour/normal pairs from the PPB/*DICER1* Registry found that, in addition to biallelic loss-of-function *DICER1* variants, frequent somatic variants were also observed in *TP53* (8/15 cases; many showed biallelic loss) and *NRAS* (2/15 cases) [28]. Another WES study found that several genes were recurrently mutated, including *TP53*, *CTNNB1*, *GPR182*, *MYH8*, *PDE2A*, and *TMX3* [27]. WES of two PPB patients with metastatic brain recurrence found pathogenic somatic variants in *TP53*, *PIK3CA, PPP2R1A* and/or *NOTCH2*, and the fusion gene *ETV6-NRTK3* [45]. Thus, human PPB tumours have a heterogeneous somatic genomic landscape, and this is mirrored in these canine cases, where there was no overlap of somatic variants between the two. The absence of any shared variants between the two species could add weight to the suggestion that PPB-like neoplasms arise via different genetic pathways in humans and dogs, or it may be that if additional cases of similar canine neoplasms can be found, more similarities may begin to emerge.

Type I PPB in humans is typically treated with surgery, with ~40% receiving adjuvant chemotherapy [46]. The current PPB/*DICER1* Registry-recommended chemotherapy is the VAC combination therapy comprising vincristine, dactinomycin (actinomycin D), and cyclophosphamide [6], which is also used to treat a range of paediatric solid tumours. A study of 118 type I PPB patients found no recurrence or progression in those who underwent adjuvant chemotherapy, with chemotherapy showing a protective association [46]. However, other studies have proposed that an intense surveillance schedule might be preferable to detect early recurrence and thus avoid adjuvant chemotherapy [47]. In contrast to type I PPB, it is recommended that type II/III PPBs be treated with surgery, intensive chemotherapy and, in some cases, radiation therapy [5]. In 2007, the PPB/*DICER1* Registry provided a protocol for a single-arm study using the IVADo chemotherapy regimen (consisting of ifosfamide, vincristine, actinomycin-D, and doxorubicin) as a frontline therapy, which resulted in similar-to-improved outcomes relative to historical controls [42]. As expected, the outcomes were worse among those presenting with metastatic disease at the time of diagnosis (most commonly to the brain and bone) [42]. In addition to IVADo, a small subset of patients also received intra-cavitary cisplatin for local control due to tumour spill [45]. It should be stated that these chemotherapy protocols are for use in humans and are presented for comparative purposes only, not to be interpreted as therapeutic recommendations for dogs.

PPB type is the strongest predictor of outcome, with 5-year overall survival (OS) rates of 100%, 98%, 83% and 62% for types Ir, I, II and III, respectively [6]. However, when type I PPB recurs, it is often as type II or III and thus has a correspondingly poor overall survival rate [47]. Local recurrence has been reported to develop in 14% of type I PPB cases and 46% of type II and III PPBs, with distant metastasis occurring in 30% of type II/III patients, primarily to the brain/spinal cord or bone [48]. Metastatic disease at the time of diagnosis of types II/III is also an independent unfavourable prognostic factor [8]. In addition, patients with extensive pleural or mediastinal involvement fare significantly worse [48]. Both dogs were managed by surgical excision alone, without adjuvant chemotherapy.

While the biological behaviour of PPB-like tumours in dogs remains uncertain, recurrence was observed in case 2. Given the very small number of cases, no association can be made between histological features, including the degree of pleomorphism, and biological behaviour. Nevertheless, this recurrence supports the value of long-term clinical follow-up in affected dogs, particularly given the recognised propensity for recurrence in human PPB.

Finally, the limitations of this study need to be acknowledged. The first is the inability to rule out some potential differential diagnoses, as the necessary diagnostic tests were not available through our laboratories, specifically *ALK* fusion evaluation and myogenin/MyoD1 IHC for IMT and PSS. The second is the use of peri-lesional tissue as the source of germline variant calls, as opposed to blood, as there is a risk of microscopic tumour infiltration/field cancerization, as well as the fact that FFPE processing introduces chemical artefacts that would not be present in DNA extracted from whole blood. The last is the lack of established diagnostic criteria for PPB in animals, making it difficult to provide a definitive diagnosis.

## 7. Conclusions

Taken together, the unusual combination of cystic epithelial-lined spaces, malignant mesenchymal proliferation with multifocal primitive morphological features and myogenic differentiation supports morphological similarity to human PPB. However, the absence of pathogenic *DICER1* variants and the histological differences observed preclude establishing direct biological equivalence. These tumours are therefore best regarded as PPB-like pulmonary neoplasms and may represent a canine counterpart of PPB, although an as-yet unclassified type of primary pulmonary sarcoma cannot be excluded.

Thus, this case report expands the limited veterinary literature on pulmonary neoplasms with PPB-like features by providing detailed descriptions of the clinical presentation, diagnostic imaging, histopathological, immunohistochemical and molecular findings, treatment, and medium-term follow-up of two dogs. In keeping with the principles of One Medicine, these cases highlight the value of comparative oncology. They also provide a basis for future comparative studies and may encourage the identification and characterisation of additional similar pulmonary tumours in dogs.

## Figures and Tables

**Table 1:** Results of the IHC performed on the two PPB-like lesions. The percentages indicate the proportion of neoplastic spindle cells showing positive immunolabelling with the respective antibody. With the pan-cytokeratin antibody, 95–100% of the non-neoplastic respiratory epithelium showed strong cytoplasmic to diffuse immunolabelling in both cases. Positive and negative controls for each antibody immunolabelled appropriately.

Antibody	Case 1	Case 2
Vimentin	70–95%, strong cytoplasmic immunolabelling	70–95%, strong cytoplasmic immunolabelling
Pan-cytokeratin	Negative	Negative
Smooth muscle actin (SMA)	5–10%, moderate to strong cytoplasmic immunolabelling	20–25%, moderate to strong cytoplasmic immunolabelling
Desmin	70–80%, mild to strong cytoplasmic immunolabelling	50–60%, mild to strong cytoplasmic immunolabelling
Synaptophysin	Negative	Negative
Chromogranin A	Negative	Negative

**Table 2:** Somatic variants identified in the PPB-like from case 1. ‘Variant location’ is relative to the CanFam3.1 reference genome, ‘protein variant’ uses HGVSp nomenclature, and ‘impact on protein’ is the predicted effect of the variant on the protein. Abbreviations: VAF, variant allele frequency.

Gene	Variant Location	Variant Class	VAF	Ensembl Transcript ID (Protein Variant)	Impact on Protein
*CRACDL*	Chr10: 43842753	In-frame insertion	0.235	ENSCAFT00000003633 (p.A605_A606insPAA)	Moderate
*DLA-64*	Chr12: 983312	Missense mutation	0.330	ENSCAFT00000036073 (p.S11P)	Moderate
*POU6F2*	Chr18: 10358917	In-frame deletion	0.167	ENSCAFT00000065823 (p.P244del)	Moderate
*TSPAN3*	Chr30: 39234646	Frameshift deletion	0.118	ENSCAFT00000079684 (p.L336Pfs*371)	High

**Table 3:** Somatic variants identified in the PPB-like from case 2. ‘Variant location’ is relative to the CanFam3.1 reference genome, ‘protein variant’ uses HGVSp nomenclature, and ‘impact on the protein’ is the predicted effect of the variant on the protein. Abbreviations: VAF, variant allele frequency.

Gene	Variant Location	Variant Class	VAF	Ensembl Transcript ID (Protein Variant)	Impact on Protein
*ADRM1*	Chr24: 46314486	Missense mutation	0.300	ENSCAFT00000020042 (p.T871P)	Moderate
*B3GAT3*	Chr18: 54023797	Frameshift deletion	0.138	ENSCAFT00000025011 (p.P183Qfs*103)	High
*GGTA1*	Chr9: 60985942	Missense mutation	0.170	ENSCAFT00000091924 (p.L64P)	Moderate
*HERPUD1*	Chr2: 59393219	Missense mutation	0.320	ENSCAFT00000046153 (p.A153V)	Moderate
*RNF138*	Chr7: 57555650	Frameshift deletion	0.500	ENSCAFT00000028621 (p.A116Ffs*8)	High

**Table 4:** Summary of the findings for both cases. Abbreviations: IHC, immunohistochemistry.

Category	Case 1	Case 2
Clinical presentation	3-year-old female neutered Labrador Retriever. Acute-onset dyspnoea and tachypnoea. Bilateral tension pneumothorax. CT: two soft tissue attenuating nodules in the right accessory lung lobe. Surgery: ruptured nodule at the tip of the accessory lobe; right accessory lung lobectomy.	1.5-year-old intact male Labrador Retriever. 2-day history of productive cough, dyspnoea and lethargy. Bilateral pneumothorax (right-sided tension) CT: two cavitary mass lesions affecting the right cranial and right caudal pulmonary lobes. Surgery: necrotic mass and ruptured bulla in the right caudal lobe, intact bulla in the right cranial lobe; right cranial and caudal lung lobectomies.
Histopathology	Well-demarcated, unencapsulated neoplasm Sheets and streams of oval to spindle cells with blastematous-to-sarcomatous morphology on a moderately abundant fibrillar collagenous stroma. Multifocal cystic structures lined by ciliated or non-ciliated cuboidal, columnar or attenuated epithelium. Moderate mesenchymal anisocytosis/anisokaryosis. Mitotic count <1 per 2.37 mm^2^ (10 × 400 HPF) Complete excision.	Moderately to poorly demarcated neoplasm. Streams and solid areas of oval to spindle cells on a loose fibrillar to dense collagenous stroma. Multifocal to coalescing cystic areas lined by ciliated to non-ciliated cuboidal to attenuated epithelium. Moderate to marked mesenchymal anisocytosis/anisokaryosis. Mitotic count was 1 per 2.37 mm^2^ (10 × 400 HPF). Necrosis and focal mineralisation. Multifocal infiltrative growth into the alveolar interstitium. Incomplete excision in the right caudal lung lobe.
IHC	Positive: vimentin, SMA, desmin. Negative: pan-cytokeratin, synaptophysin, chromogranin A.	Positive: vimentin, SMA, desmin. Negative: pan-cytokeratin, synaptophysin, chromogranin A.
Molecular profile	Somatic mutations in: *CRACDL*, *DLA-64*, *POU6F2*, *TSPAN3*. No potential disease-causing germline *DICER1* variants identified.	Somatic mutations in: *ADRM1*, *B3GAT3*, *GGTA1*, *HERPUD1*, *RNF138*. Manual inspection of *DICER1* sequencing data identified a possible somatic mutation (C>T) of low VAF (3%), so the possibility of an FFPE sequencing artefact cannot be excluded. No potential disease-causing germline *DICER1* variants identified.
Follow up	Free of clinical signs at 20 months post-surgery.	Almost 2 years after initial surgery, a pulmonary lesion arising from a remnant of the previously resected right cranial lung lobe; identified and removed. Free of clinical signs 4 months after the second surgery.

## Data Availability

The data presented in this study are openly available in European Nucleotide Archive at https://www.ebi.ac.uk/ena/browser/view/ERP179949, reference number ERP179949.

## Supplementary Materials

The following supporting information can be downloaded at https://www.mdpi.com/article/10.3390/vetsci13101111/s1, Table S1: Germline loss-of-function variants of unknown significance identified in case 2.

## Abbreviations

The following abbreviations are used in this manuscript:

CT	Computed tomography
EXPeRT	European Cooperative Study Group for Pediatric Rare Tumors
FFPE	Formalin-fixed, paraffin-embedded
H&E	Haematoxylin and eosin
IHC	Immunohistochemistry
IMT	Inflammatory myofibroblastic tumour
IVADo	Ifosfamide, vincristine, actinomycin-D, and doxorubicin
OS	Overall survival
PPB	Pleuropulmonary blastoma
PSS	Pulmonary synovial sarcoma
T-FAST	Thoracic Focused Assessment with Sonography for Trauma/Triage
VAF	Variant allele frequency
WES	Whole-exome sequencing
WHO	World Health Organisation
